# Evaluation of comprehensive early warning for higher education institutions’ cloud model of simulated enterprise management cockpit

**DOI:** 10.1371/journal.pone.0305652

**Published:** 2024-06-18

**Authors:** Weixing Fan, Zhifeng Li

**Affiliations:** Department of School of Accountancy, Guangzhou Huashang College, Guangzhou, China; Al Mansour University College-Baghdad-Iraq, IRAQ

## Abstract

The cross-disciplinary virtually simulated platform for enterprise management in universities’ new business courses is an initiative practical framework based on scenario-driven tasks. However, there is a prominent conflict between the rapid operating cycle of simulation enterprises plus their fierce competitions and the strategic demand for real-time analysis for operational data. Based on such demand, this study takes the development method of the simulated enterprise management cockpit from Guangzhou Huashang College as an example. It adopts the combined weighting method based on cloud models to determine indicator weights, then qualitative and quantitative data analyses are conducted from five aspects: “business, finance and operation”, “customer management and marketing”, “internal operational objectives”, “product development strategy”, along with “team building and management”. This approach achieves a comprehensive evaluation and early warning of the enterprise management process. Specifically, the subjective weights are determined by the Analytic Hierarchy Process, while the objective weights by the entropy weight method, finally verified by cloud model evaluation of its overall indicator performance. The design can evaluate the comprehensive performance of enterprise management indicators and students’ activity participation through the cloud-based application and the digital cockpit, so as to fully presents the enterprise’s overall management level, along with judgement of whether it is reasonable through pointers in different colors. In addition, apparent indicator-related characteristics are also utilized to assess future decision-making directions. Finally, this comprehensive approach can timely optimize operation strategies and facilitate budget allocation for future development.

## 1. Introduction

### 1.1 Background

In response to fierce competitions in current business environment, enterprises surely need swift and precise data collection and analysis for decision-making and performance monitoring. In this context, the enterprise management cockpit as an integrated information platform has developed into a powerful tool for the management to better understand current enterprise status and trends. This study centers around the pivotal role of such cockpit in promoting efficient management practices through discussions of its design and implementation, together with relevant advice accordingly.

The National Education Work Conference in 2023 proposed to fully implement the digital education through national strategies, so as to inject new vitality to educational development [1]. More specifically, major tasks in 2023 for the Ministry of Education’s Higher Education Department can be summarized as “further promotion of ‘Four News’ (new majors, institutions, models, and paradigms)” along with a new step forward in independent talent cultivation. Furthermore, disciplinary integration is highlighted, featured by the development of new liberal arts, quality and organizational model innovations [2].

To this end, followed by the idea of new business, business schools with management-related disciplines have embraced the new education paradigm to cultivate talents in comprehensive cross-disciplinary enterprise simulation courses, thus addressing the inadequate support for higher operational efficiency of simulated enterprises. Traditionally, such issue is primarily manifested by the conflict between the complexity and urgency of the operational process and the guidance for value enhancement in simulated enterprises, along with the mismatch between the training for decision-makers and their actual performance. In addition, the simulation process highly needs a cloud-based evaluation and analysis tool that coordinate with overall enterprise operation. This tool can facilitate real-time integration of and insight into operation data and team-building evaluations. In addition, early warning through visible meters are available for decision-makers to rapidly assess their own capabilities, then realize efficient decisions and agile management.

**Fig 1:**
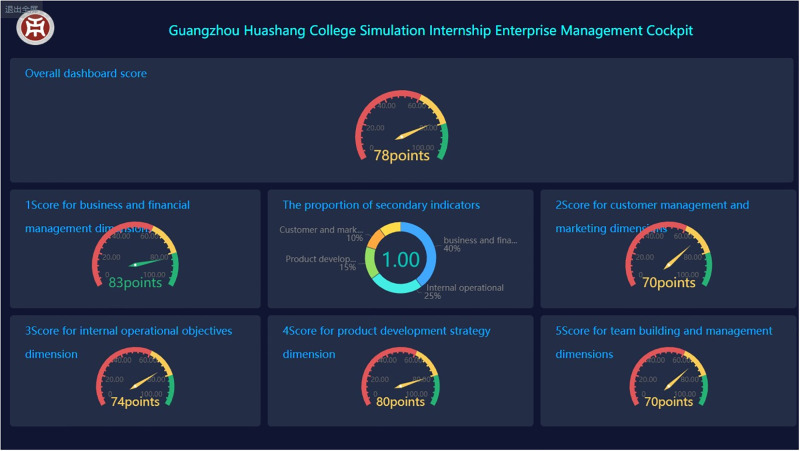
Guangzhou Huashan College’s simulation intern enterprise management cockpit.

**Fig 2:**
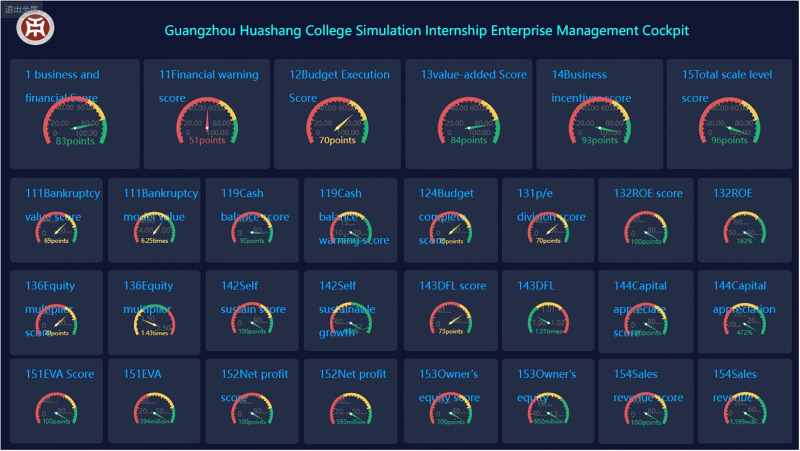
Example of the segmented meters in the simulation intern enterprise management cockpit from Guangzhou Huashang College.

Correspondingly, the simulated enterprise management cockpit from Guangzhou Huashang College is adopted in this study. By studying the indicators’ design system for the cloud-based simulated intern enterprise management cockpit, a combined weighting method based on cloud models can be utilized to integrate qualitative and quantitative data evaluation. Specifically, real-time quantitative data comes from databases, while timely qualitative data from robot collection of the enterprise’s operational processes and evaluation results of team-building activities. Then, the weighting algorithm is employed to evaluate relevant data by different levels and corresponding results are presented visually. Through timely diagnosis and early warning provided on the cloud platform, decision-makers can optimize their management, as shown in Figs 1 and 2.

In summary, considering current trends in information technology and management disciplines, it is of practical significance to deal with the building of the early warning evaluation system for the simulated enterprise management cockpit in higher education institutions from the perspective of cloud model and combined weighting, furthermore in promoting the visual methods of enterprise management empowered by data intelligence. To be noted, the simulated enterprise management cockpit from higher education institutions will be hereinafter referred to as “the enterprise management cockpit”, and Abbreviated as “EMC”.

The importance of this research of simulated enterprise management cockpit in this paper: (1) constructing a comprehensive operational safety evaluation index system, and comprehensively and scientifically evaluating the operating status and development trends of the enterprise through the combination weight method; (2) Through advanced data visualization and information technology, the core business and financial indicators of the enterprise are presented intuitively, enabling managers to respond quickly and combining macro and micro indicators to timely identify potential risks and achieve accurate decision-making; (3) By applying the principles of cloud models, it is possible to provide real-time feedback on the warning levels of enterprises under different operating conditions, provide targeted warning information, and help prevent and respond to potential business risks in a timely manner.

### 1.2 Research meaning of the early warning evaluation system for the EMC

#### 1.2.1 Theoretical implications

This study aims to address the shortcomings of talent cultivation methods in economic management disciplines, align with platform based big data technology, practice new business education concepts, innovate cross disciplinary enterprise simulation comprehensive practice talent cultivation methods, and break through the bottleneck of traditional platform based talent cultivation methods in supporting the operational effectiveness of simulation enterprises. On the one hand, the theoretical significance of this study lies in solving the problems of structuring, systematizing, and scientificity of enterprise decision-making information. It can be an evaluation and analysis tool that fits the panoramic view of enterprise operation, and can integrate and perspective enterprise operation process data and team building activity evaluation results in real time; On the other hand, the theoretical significance of this study lies in whether the corresponding information of decision-making positions, such as CEOs, CFOs, and CMOs, matches the actual functional data needs of the positions; Finally, the theoretical significance of this study lies in its ability to provide instrument based warning support, distinguishing warning levels through color or indicator values, assisting enterprise decision-makers in quickly measuring their own capabilities, efficiently predicting decisions, and achieving agile management.

#### 1.2.2 Practical implications

Apart from the object in this study, investigations and exchanges have been conducted with similar simulation platform courses at Guangdong University of Finance and Guangdong Baiyun University. According to feedbacks, in the building of cross-disciplinary and comprehensive intern simulation platforms in higher education institutions, cloud management mobile services should be integrated. Moreover, a scientific and reliable evaluation framework for management cockpit system should be fully utilized to support informatization decisions. Finally, both physical wall-mounted panel displays and synchronized queries on mobile terminals are available to enterprises’ digital management of strategic performance.

Therefore, relying on the current application status of the simulation platform at the School of Business of Guangdong University of Finance, there is an urgent need for objective alignment of talent cultivation, together with collective and timely feedback on simulation effects. So that intern traces and competency levels of respective majors and positions are visually clearer, further highlighting the significance of applying this approach in teaching management of business majors in the modern era.

### 1.3 Research methodology

#### 1.3.1 Literature research method

Firstly, it is necessary to collect research, theoretical and practical significance, as well as domestic and foreign research reviews on decision support systems and information system evaluation. By summarizing and summarizing previous research results, the research ideas and methods of this article can be obtained. Based on the reference of previous research results, further research can be conducted.

#### 1.3.2 Case study method

The case analysis method requires a full understanding of the actual situation of the case enterprise. Only by comprehensively understanding the situation of the case enterprise can the conclusions drawn have credibility and persuasiveness, and reach conclusions with high credibility. This conclusion will be universal, deepen the understanding and interpretation of similar events, and provide a certain reference for the development path of similar enterprises.

Through case analysis and research on the design and application process of the management cockpit system at Guangdong University of Finance and Economics Huashang School, the key factors affecting the success of the project and the focus of subsequent optimization are summarized.

#### 1.3.3 Inductive summary method

Through the digital and intelligent reform of efficient talent cultivation, the big data teaching concept and teaching methods of economic and management simulation platforms, and the collection of data related to enterprise management cockpit, enterprise performance management, financial analysis, and other aspects in recent years, more objective theoretical and practical application support are selected.

## 2. Literature review

### 2.1 Literature review of international researches

In terms of modern enterprise management, the theory of Business Intelligence (BI) has developed from data warehouse to Balanced Scorecard (BSC), further evolving into the panel-style management tool known as “management cockpits” supported by visual virtual information technology. Through this approach, an integrated strategic performance system can be built for a visual and comprehensive management application that covers the whole picture and details, thus supporting all decision-making by multidimensional indicator dashboards as follows:

First, the current status of international BI researches. The preliminary BI theory, including data warehouse, online analytical processing and data mining, is rooted in transforming existing data within enterprises into knowledge, thereby contributing to informed business operational decisions [3].

The next step forward primarily in the 1990s mainly developed in databases and data modeling, with the new concept of directed graph workflow mining models—data mining [4].

More recent advancements in this aspect refer to the theory of intuitive visualized management, namely the dashboard management theory. Thereby, relevant intelligent analytical concepts are more clear, including visual dashboard analysis, control and self-service analysis, integrated data product chains, cloud-based management data analysis, mobile terminal analysis and IoT data analysis [5].

Moreover, the rise of service-oriented personal cloud computing requires new technologies for asset discovery, exchange, and management. In response, a large-scale distributed system on unified personal clouds is then proposed, namely interactive cockpits that manage personal clouds and their alliances. In this way, there is less reliance on central infrastructure, while greater balance between service supply and consumption [6].

To deal with cross-functional communication and collaborative management within the management cockpit, a control and management process is established based on intellectual property and strategic orientation. This approach has been proved successful within enterprises and interdisciplinary management teams, enabling structural, categorized, and managing functionsv—with abundant visualized information along with extensive analysis and simulation options for comprehensive and sustainable performance management [7].

In terms of BSC’s application in higher education, the recommended method is to formulate strategies for implementing BSC within educational institutions, then apply and observe its effectiveness in organizational assessments. The aim is to define a management and tracking system for industry bench-marking [8].

During the strategic implementation phase, the management proposes to interpret strategic execution as a dynamic decision-making task. Learning from previous experience of solving complex problems and performance measurement, more explorations are conducted on whether factors such as BSC cockpit, intelligence, and knowledge can explain variations in strategic execution performance [9].

When considering differentiated decisions of implementing strategies and outcomes resulting from various cockpits, scholars proposed to integrate ideas related to strategic operations. Therefore, they design a closed-loop control task for strategic implementation and advocate a BSC strategy map cockpit. This cockpit provides participants with a concentrated and useful interface with both theoretical and practical contributions during implementation, also facilitating the modeling considering the impact of management performance measurements [10].

Second, The current international research status of evaluating enterprise management information systems. So far, popular evaluation methods for information systems worldwide include qualitative expert analysis using the Delphi method, fuzzy composite evaluation based on fuzzy matrices, the quantification of qualitative data through a hierarchical quantitative analysis using the Analytic Hierarchy Process, multi-criteria decision analysis, along with expectation models that emphasize customer satisfaction. Specifically, ideas on strategic performance evaluation are as follows:

In terms of performance management, relevant theories proposed to integrate an enterprise’s overall strategy with its financial indicators and non-financial information. To be noted, the crucial connection between overall strategy and performance indicators during evaluation part is fully highlighted, therefore a Performance Pyramid model is designed accordingly [11].

In addition, the reform-centered view of performance evaluation reveals enterprises’ sustained development capability, which is theoretically sophisticated, but rarely applied in practice [12].

The financial management system EVA (Economic Value Added) came out, which combines decision-making, incentive, and performance evaluation. In particular, it emphasizes enterprises’ costs of all capital inputs and their long-term development [13].

Serving as a performance assessment system, the BSC is of great significance in implementing enterprise strategies. It provides a framework that focuses on key management processes, assessing an enterprise’s past performance, together with its future development potential and competitiveness [14].

When evaluating enterprise performance, the “Four-Dimension” framework proposed by Robert Hall is adopted, including quality, lead time, resource utilization, and human resource development. These four non-financial indicators are included into the performance evaluation system and improvements in any of these dimensions should not sacrifice others, so as to reduce competitive risks.

Regarding decision support systems, the solution is to integrate visualize method and decision modeling capabilities with human insights and interaction abilities, thus developing a decision support system known as Forest Community-DSS. By leveraging techniques like DT (Decision Theater), FC-DSS and relevant technologies can enhance stakeholder involvement in decision-making through higher participation frequency, more input from stakeholders, support given to the development and evaluation of alternative solutions, and optimized selection of preferential alternatives, as highlighted by Boukherroub, D’amours, & Rönnqvist [15].

Third, Overseas research status of management cockpit systems. Among developed countries, the management cockpit system serves as sophisticated decision support within information systems, which demonstrates an advanced development stage of business intelligence systems that evaluated by the fourth-generation BSC concept. This system has now been widely adopted in various industries, with verified values and efficiency in enterprise competition and operations.

So far, prominent global suppliers of such cockpit information system mainly refer to SAP, Oracle, Microsoft, SAS, Qlik, Tableau and Microstrategy, basically monopolies in sectors such as telecommunications, finance, retail, and insurance.

### 2.2 Literature review of research status in China

In China, domestic researches on evaluating enterprise performance primarily focuses on local applications of advanced international models. Apart from the characteristics of Chinese enterprise s’ supply chains, scholars adopted a hierarchical approach based on BSC theory. They emphasize the need for comprehensive evaluations considering multiple factors, thus upgrading the indicator system that integrates BI theory.

First, the domestic BI research status. The initial BI research in China dates back to a relevant report in 1998, presenting several views discovered in the management community. After that, theoretical researches in specific areas emerged, such as starting from interpreting BI concept from management, technology, and application perspectives [16], then corresponding implementation strategies [17], and further control recommendations during BI implementation [18].

The cloud technology and business intelligence was integrated, featured by resource sharing, low costs, and real-time interactions [19]. In addition, a system dynamics model for strategic performance evaluation was introduced, which effectively combines relevant theories and research methods of BSC and system dynamics. In detail, this model had an extra BSC dimension of social responsibility, finally building a dynamic five-dimensional model with an indicator system and strategic map for evaluating enterprise strategic performance by simulation software [20].

To upgrade agile enterprise management, a management cockpit was proposed to integrate various business units and break down the barriers between departments. This efficient method functions well in handling with huge amounts of isolated cross-organizational business data at different management levels, thus mitigating “information silos” and enhancing the enterprise’s competitive prowess in dynamic markets [21]. What’s more, a multi-dimensional intricate system of budget analysis indicators was developed to support lean management and facilitate feedback. Particularly, the management cockpit works as a budget compass and baton, directing towards efficient resource allocation and continuous improvement [22].

Second, the domestic research status of information system evaluation. Despite being not mature, relevant domestic researches on information system evaluation have already yielded significant results. One previous study proposed an evaluation system for enterprise informatization, featured by 20 primary indicators and specific calculation methods. This study was also a pioneer in measuring the effectiveness of enterprise informatization by quantifying the correlation between the level of informatization and enterprise performance [23].

After several years of technology advances, a comprehensive life-cycle evaluation system was put forward, which utilized the hierarchical grey method and verified its scientific validity and applicability through case study [24].

In order to further upgrade the evaluation system, user-friendly idea prevailed, specifically valuation metrics of relevant users in different stages were established, including researches on the evaluation indicator system for building enterprise information system considering both enterprise management personnel and front-line operators [25].

Based on the multi-dimensional utility theory, scholars introduced a flexible information system evaluation model accordingly in multi-objective decision-making. Supported by a comprehensive and objective indicator system, a hierarchical structure for information system evaluation utilizing a multi-dimensional utility model, along with specific merging rules and weights, the overall effectiveness of information systems has been fully validated [26].

Among various relevant evaluation methods, scholars suggest a combination of two or more methods [27].

Then, scholars proposed an enhanced value evaluation model according to stakeholder theory. This model combines both financial and non-financial indicators in three dynamic dimensions that reflects the organization’s value and strategic performance, namely growth performance, profitability performance, and risk performance [28].

From the perspective of cultivating students’ innovation and entrepreneurship capabilities, the dashboard can be utilized, so that a pragmatic virtual simulation teaching platform can visualize relevant resources based on business management disciplines. In this way, traditional low efficiency in transferring theoretical knowledge into practical skills is no longer a problem. Instead, this method can motivate an active entrepreneurial mindset and participation, so as to enhance the entrepreneurial qualities of high-end management professionals [29].

Furthermore, through the above literature review research, the performance of business functions in evolving into intelligent enterprise management cockpit management services lies in: (1) the ability to utilize technologies such as big data, cloud computing, artificial intelligence, and automation to integrate ERP databases with other systems; (2) Can provide agile, scenario based, and intelligent management and decision-making services for enterprises, help them improve their digital capabilities, and achieve maximum data value; (3) It has the characteristics of low code, light configuration, easy operation, strong support, open integration, compatibility with multiple platforms, and emphasizes data security and privacy protection. In summary, this enterprise management service can provide rich and multi-dimensional custom data display, report visualization, and interaction capabilities, helping enterprises better manage and analyze data, making wiser decisions, and emphasizing data security and privacy protection.

## 3. Overview of relevant theories

### 3.1 Enterprise management cockpit

The enterprise management cockpit is a management information center system that provides "One Stop" decision support for senior management. It visually displays the key indicators (KPIs) of enterprise operation in the form of a cockpit, through various common charts (speedometer, volume bar, warning radar, and radar), intuitively monitors the operation of the enterprise, and can provide early warning and mining analysis of abnormal key indicators.

When enterprise managers have no time to query in the physical cabin, they can synchronously query the corresponding dial data with mobile phones and other mobile terminals under Internet conditions, so as to achieve an integrated online and offline application.

The management cockpit incorporates the management philosophy of the balanced scorecard, which unfolds the company’s vision and strategy through a strategic map layer by layer into a set of dynamic and measurable KPI indicators. The internal processes and team learning aspects emphasized by the balanced scorecard correspond to the blue wall content of the management cockpit, the business, finance, and operation aspects correspond to the black wall content of the management cockpit, and the product development and customer aspects correspond to the red wall content of the management cockpit;

### 3.2 Analytic Hierarchy Process

Analytic Hierarchy Process (AHP) is a qualitative and quantitative decision-making method proposed by Professor Sati in the early 1970s in the United States. Its characteristic is to conduct in-depth analysis of the essence, influencing factors, and internal relationships of complex decision-making problems, and on this basis, use quantitative information to mathematize the thinking process of decision-making, solve complex decision-making problems with multi-objective, multi criteria, or unstructured characteristics, thereby making decision-making methods more convenient. This method decomposes into different hierarchical structures in the order of the overall goal, sub goals at each level, evaluation criteria, and specific backup plans. Then, the method of solving the judgment matrix eigenvectors is used to obtain the priority weights of each element in each level for a certain element in the previous level. Finally, the method of weighted sum is used to gradually merge the final weights of each backup plan for the overall goal. The one with the highest final weight is the optimal plan.

In modern decision-making assistance applications, with the help of information technology, the elements related to decision-making are decomposed into levels such as goals, criteria, and plans through matching. The network system theory and multi-objective comprehensive evaluation method are applied to propose a hierarchical weight decision analysis method.

The management simulation platform of the Huashang School of Guangdong University of Finance and Economics is facing a panoramic simulation of socialized operations. In the face of centralized data at various levels, it is necessary to determine the impact weights and select the best ones, in order to select key indicator data that can affect operations.

### 3.3 Fuzzy comprehensive evaluation method

This comprehensive evaluation method transforms qualitative evaluation into quantitative evaluation based on the membership theory of fuzzy mathematics. The basic principle is: first, determine the set of factors (indicators) of the evaluated object and evaluate the set of levels; Secondly, determine the weights and membership degree vectors of each factor separately to obtain a fuzzy evaluation matrix; Thirdly, perform fuzzy operations on the fuzzy evaluation matrix and the weight vectors of factors, and normalize them to obtain the fuzzy comprehensive evaluation results.

The management simulation platform of Huashang School of Guangdong University of Finance and Economics requires enterprises to objectively reflect the actual situation of their business processes, including the assessment of their own human capital, timely feedback on their business operations, and business dealings with peripheral institutions such as industry and commerce, taxation, and auditing. Both quantitative and qualitative data are needed, and they need to be unified into a quantitative caliber. Therefore, modern technology and fuzzy mathematics are needed to integrate them together.

### 3.4 Cloud model theory

Cloud model theory is a combination evaluation method that combines qualitative knowledge with quantitative data to integrate uncertain data. It achieves quantitative description of concepts through the concept of clouds. The theoretical principle is that clouds are composed of many cloud droplets, each representing a possible value and its membership degree. This theory introduces probability theory and fuzzy set theory, and its advantage lies in dealing with uncertainty and fuzziness problems, especially in fields such as data mining, intelligent control, and decision support. It provides an effective tool and method for handling uncertain information, improving the accuracy and adaptability of decision-making.

## 4. Material and methods

### 4.1 Re-building of the early warning evaluation system for the EMC in higher education institutions

Considering a new competitive environment and the strategic evaluation needs for enterprises’ scale development, higher education institutions should highlight that the development of management practices is the condition for modern enterprises to employ new performance evaluation methods. In addition, comprehensive factors should be taken into consideration, including those aspects related to enterprise development goals, organizational structure, organizational capabilities, comprehensive budget management, incentive mechanisms, indicator quantification, internal controls, and financial analysis. Moreover, based on the core BSC ideas of enterprise performance evaluation, scientific methods of fuzzy judgment and analytic hierarchy process (AHP) were adopted to determine each indicator weight in the comprehensive evaluation function model. Finally, beyond operating as a teaching method for stronger enterprise management, this approach also contributes to a new regional/ global digital decision system.

More specifically, the first step is to cultivate relevant abilities and confirm board performance, then the next to optimize indicator selection combining the characteristics of simulation enterprise on the platform and expert opinions, so as to develop a comprehensive evaluation indicator system for early warning based on cause-and-effect relationship chain and strategic mapping. To be noted, the target, or the enterprise’s strategic goals, was initially divided by AHP into four layers from top to bottom. In detail, the first layer represents the comprehensive early warning evaluation layer (the comprehensive layer), showing the overall dashboard performance. Then this layer was further sub-divided into five specific strategic decision layers (the first-level dimensions), namely the dimensions of business, finance, and operation, customer management and marketing, internal operational objectives, product development strategies, along with team building and management. Furthermore, each strategic decision layer was broken down into tactical operational layers (the second-level dimensions), which further divided into specific management execution indicators (the third-level dimensions). Particularly, the relevance of each indicator to the indicator system was identified, finally shaping a four-layer comprehensive evaluation indicator system featured by a “one overall and three-dimensional” framework (as shown in Table 1).

**Table 1:** Comprehensive early warning evaluation indicator system of simulated enterprise management cockpit from higher education institution.

Comprehensive layer	First-level dimensions	Second-level dimensions	Third-level indicator dimensions and correlation	
Overall dashboard U	Business, finance, and operation dimension U1	Financial warning U11	Bankruptcy warning model value U111	Positive
			Debt coverage ratio U112	Positive
			Asset return rate U113	Positive
			Debt-to-equity ratio U114	Negative
			Funds safety ratio U115	Positive
			Core profit margin U116	Positive
			Working capital turnover rate U117	Positive
			Overall target profit achievement U118	Positive
			Cash balance warning U119	Positive
			Cash coverage ratio U1110	Positive
			Sales cash ratio U1111	Positive
		Budget execution deviation U12	Budget accuracy rate U121	Positive
			Annual budget completion rate U122	Positive
			Annual budget deviation rate U123	Negative
			Quarterly budget completion rate U124	Positive
			Quarterly budget deviation rate U125	Negative
		Business value-added efficiency U13	P/E ratio difference rate U131	Positive
			ROE (Return on Equity) U132	Positive
			Return on total assets U133	Positive
			Return on capital U134	Positive
			Capital accumulation rate U135	Positive
			Equity multiplier U136	Negative
			Cost-to-profit ratio U137	Negative
		Operational incentive quality U14	Operating profit growth rate U141	Positive
			Self-sustaining growth rate U142	Positive
			Financial leverage coefficient U143	Negative
			Capital preservation and appreciation rate U144	Positive
			Main business profit margin U145	Positive
			S minimum share Q sales proportion U146	Positive
		Scale and total level U15	Economic value added U151	Positive
			Net profit U152	Positive
			Owner’s equity U153	Positive
			Sales revenue U154	Positive
			S’s Q quantity U155	Positive
	Customer management and marketing dimension U2	Customer focus ratio U21	Main customer retention rate U211	Positive
		Key customer segmentation U22	Main customer profitability rate U221	Positive
		Customer satisfaction level U23	Customer satisfaction rate U231	Positive
	Internal operation objective dimension U3	Business warning U31	Purchase order completion rate U311	Positive
			Enterprise scale U312	Positive
			Break-even point U313	Negative
			Product stability and volatility U314	Positive
			Price changes of products U315	Negative
			Input factor price changes U316	Negative
			S operating leverage coefficient U317	Negative
			Trends in quarterly purchase amount U318	Positive
			Sales order completion rate U319	Positive
			Overall purchase amount ratio U3110	Negative
		Operational support factors U32	Total asset turnover rate U321	Positive
			Inventory turnover rate U322	Positive
			Current asset turnover rate U323	Positive
			Accounts receivable turnover rate U324	Positive
			Days sales outstanding U325	Negative
			Days inventory outstanding U326	Positive
			Fixed asset turnover rate U327	Positive
			Accounts payable turnover rate U328	Negative
			Current liability ratio U329	Negative
			Long-term liability ratio U3210	Positive
			Equity ratio U3211	Positive
			Current asset utilization rate U3212	Positive
			Fixed asset utilization rate U3213	Negative
			Other long-term asset utilization rate U3214	Negative
			Inventory-to-total assets ratio U3215	Negative
			Non-performing asset ratio U3216	Negative
		Risk prevention performance U33	Audit report opinion type U331	Positive
			Working capital U332	Positive
			Current ratio U333	Positive
			Quick ratio U334	Positive
			Debt-to-equity ratio U335	Negative
			Ownership ratio U336	Negative
			Total leverage coefficient U337	Negative
			Fixed charge coverage ratio U338	Positive
			Cash ratio U339	Positive
			Cash flow ratio U3310	Positive
			Operating cycle U3311	Negative
		Internal operational efficiency U34	Sales revenue growth rate U341	Positive
			Net profit growth rate U342	Positive
			Total assets growth rate U343	Positive
			Fixed assets growth rate U344	Positive
			Per capita profit U345	Positive
		Compliance management level U35	Number of major defaults U351	Negative
			Number of general defaults U352	Negative
	Product development strategy dimension U4	Overall product benefits U41	Gross profit margin U411	Positive
			Net profit margin U412	Positive
			Advertising expense to sales revenue ratio U413	Negative
			Proportion of revenue from new products U414	Positive
			Changes in inventory-to-revenue ratio U415	Negative
			Expenses-to-revenue ratio U416	Negative
			Technology investment ratio U417	Negative
		Product structure composition U42	Low/mid/high-grade product output ratio U421	Positive
			Low/mid/high-grade product quantity ratio U422	Positive
	Team building and management dimension U5	Team overall satisfaction U51	Employee satisfaction rate U511	Positive
		Team organizational level U52	Execution ability U521	Positive
			Senior staff recruitment rate U522	Positive
			Key employee turnover rate U523	Negative
			Corporate brand and culture display construction U524	Positive
		Learning organization capability U53	Organizational competency performance U531	Positive
			Organizational learning and growth capability U532	Positive
			Employee training rate U533	Positive

This study adopts a three-level indicator system to identify the positive and negative correlation of specific indicators at the third level. (1) Positive correlation indicates that the larger the indicator value is; the higher utility the indicator system is. (2) Negative correlation represents the opposite.

Specific indicators for business and finance include: (1) Financial warning, which measures operational risks from the financial perspective. (2) Budget implementation deviation, which measures the level of financial control in terms of budget effectiveness. (3) Business value-added efficiency, which reflects the value of investment to shareholders. (4) Operational incentive quality, which measures the operational efficiency from the financial perspective. (5) Level of total scale, which reflects the operational status in quantitative ways.

Specific indicators for customer management and marketing include: (1) Proportion of attention customers, which analyzes the contribution of different types of customers. (2) Key customer segmentation, which explains the effectiveness of management on important customers. (3) Customer satisfaction, which reflects to what extent products or services satisfy consumers.

Specific indicators for internal operational objectives include: (1) Business warning, which measures operational risks from a business perspective. (2) Operational support, which reflect a company’s operation efficiency in terms of turnover. (3) Risk prevention performance, which shows the effectiveness of social auditing, debt levels, and operational conditions from a view of risk control. (4) Internal operational efficiency, which shows management effectiveness according to a company’s development efficiency. (5) Compliance management level, which measures the level of management compliance with legal and ethical standards of a company.

Specific indicators for product development strategy include: (1) Overall product benefits, which reflects the contribution of product value. (2) Product structure, which exhibits the product mix from the perspective of quantity and value.

Specific indicators for team building and management include: (1) Team overall satisfaction, which values employees’ satisfaction on the company’s working environment. (2) Team organizational level, which measures the effectiveness of organizational management within the company. (3) The ability of being a learning organization, which reflects the progress of human capital.

### 4.2 Theory for determining the weight of comprehensive early warning evaluation indicators for the EMC

#### 4.2.1 Subjective weights determined by AHP

**Fig 3:**
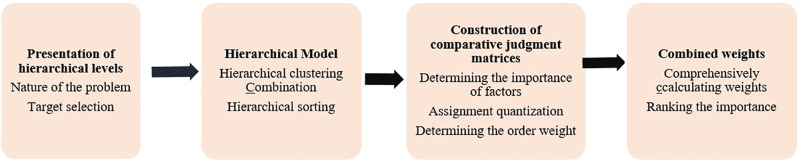
Weight determination paths by AHP.

The Analytic Hierarchy Process (AHP) is a comprehensive analysis method integrating qualitative and quantitative analysis. Proposed by Professor A.L. Saaty of the University of Pittsburgh in the 1970s, this method applies network system theory to solve multi-objective comprehensive evaluation problems. By assigning weights to decision-related indicators in a hierarchical order, it provides a clear and straightforward solution to the ranking of multi-objective, multi-criteria, and multi-level factors. Specific implementation process is described in Fig 3.

*4*.*2*.*1*.*1 Defining the hierarchical structure model for the set of indicators*. According to Table 1, the comprehensive early warning evaluation indicator system proposed by this study is structured as follows. The top-level is the objective layer, denoted as U. The first-level indicators are known as *U*_1_, *U*_2_, …, *U*_*m*_. The second-level indicators are recorded as *U*_11_, *U*_12_, …, *U*_*mn*_. The third-level indicators are denoted as *U*_111_, *U*_112_, …, *U*_*mnt*_.

*4*.*2*.*1*.*2 Constructing comparative judgment matrices*. Firstly, a judgment matrix A is constructed as (*a*_*ij*_)_*m*×*m*_ for each hierarchical level corresponding to the three dimensions in Table 1. Then, technical experts and scholars are invited to compare the importance of each pair of indicators within the hierarchical level with a scale of 1 to 9 to determine the judgment matrices for each level.

*4.2.1.3 Normalizing the judgment matrices at each level and obtaining the normalized vector w_i_ of the i-th indicator*.


$$w_{i}=\sqrt[{m}]{a_{i1}a_{i2}a_{i3}\cdots a_{im}}$$ (1)

*4.2.1.4 Determining the weight coefficient of each indicator by weighting the judgment matrices at each level*.


$$\omega_{i}=\frac{w_{i}}{\sum_{1}^{m}w_{i}}$$ (2)

*4.2.1.5 Calculating the maximum eigenvalue λ_max_ of judgment matrix with MMULT function and checking the consistency of CR*.


$$\lambda_{max}=\sum_{1}^{m}\frac{{\left(A\omega \right)}_{i}}{n\omega_{i}}$$ (3)

Calculating the consistency index CI

$$CI=\frac{\left(\lambda_{max}-n\right)}{n-1}$$ (4)

When CI = 0, it shows that there is consistency in the judgment matrix, and the larger the value is, the worse the consistency is.

By looking up the table, we can get the average random consistency index RI.


$$CR=\frac{CI}{RI}$$ (5)

When CR≤0.1, the judgment matrix has an acceptable degree of consistency as needed, otherwise it should be adjusted.

#### 4.2.2 Determining the objective weight based on entropy weight method

The entropy weight method, put forward by Shannon in the 1940s, is used to determine the weight based on the dispersion of objective data. Weights are assigned to indicators according to the information entropy formula, and then adjusted to ensure objectivity.

*4*.*2*.*2*.*1 Normalizing the third-level indicator matrix for each second-level indicator*. The second-level indicators are classified into positive and negative ones according to the attributes of their relevant third-level indicators as shown in Table 1. For positive indicators, the higher score the comprehensive evaluation result is, the more the indicator contributes to the one at a higher level. For negative indicators, the higher score the comprehensive evaluation result is, the less the contribution it makes. As the third-level indicators under the second-level ones have different measurement, it is necessary to standardize them to ensure comparability. The specific approach is as follows: first, the absolute values of indicators are converted into relative values to avoid the influence of dimensional difference on evaluation results. By doing so, normalized values would fall within the range of [0, 1]. The indicator system should be normalized using the min-max method. The positive indicator is applied with Eq (6) and negative one, Eq (7). The formulas are as follows:

$$x_{ij}^{\prime }=\frac{x_{ij}-x_{min}}{x_{max}-x_{min}}$$ (6)

$$x_{ij}^{\prime }=\frac{x_{max}-x_{ij}}{x_{max}-x_{min}}$$ (7)

Where,

*x*_*j*_ = the value of the j-th indicator;

x_max_ = the maximum value among different evaluation series of the same indicator;

*x*_*min*_ = the minimum value among different evaluation series of the same indicator.

*4*.*2*.*2*.*2 Data shift*. In case meaningless data such as zero and negative values affect the calculation of subsequent data, this study adopts a data shift strategy to resolve this problem, by adding a correction value *α* to each normalized datum.


$$x_{ij_{1}}^{\prime }=x_{ij}^{\prime }+\alpha ,\alpha =1*10^{-6}$$ (8)

*4.2.2.3 Determining the weight **y**_ij_ of the third-level indicators under corresponding second-level indicators*.


$$y_{ij}=\frac{x_{ij1}^{\prime }}{\sum_{i=1}^{m}x_{ij1}^{\prime }}\left(1\ll y_{ij}\ll 1\right)$$ (9)

*4*.*2*.*2*.*4 Calculating the j-th information entropy value* ***e***_***j***_ *for each indicator*.

$$k=\frac{1}{lnm}$$ (10)

$$e_{j}=-k*\sum_{i=1}^{m}y_{ij}ln_{y_{ij}}$$ (11)

Where, K is a constant determined by the number of samples.

*4.2.2.5 Calculating the j-th information utility value **d**_**j**_ for each indicator*.


$$d_{j}=1-e_{j}$$ (12)

The information utility value for a particular indicator, denoted as *x*_*ij*_, depends on the difference between 1 and the information entropy value *e*_*j*_ of that indicator. This information utility value directly influences the weight assigned to the indicator *x*_*ij*_. A higher information utility value indicates greater importance of the indicator and such indicator is assigned with a high weight.

*4.2.2.6 Calculating the weight **w**_**j**_ for the third-level indicators under the corresponding second-level indicator*.


$$w_{j}=\frac{d_{j}}{\sum_{i=1}^{m}d_{j}}$$ (13)

#### 4.2.3 Determining the weight mix for the third-level indicators under the corresponding second-level indicators

The weight mix Wi for the third-level indicators under the corresponding second-level indicators can be calculated as: subjective weights determined by the AHP multiply the proportion of the weight mix α, and objective weights determined by the Entropy Weight Method multiply the proportion (1-α). The process is as follows:

$$Wi=\alpha \omega_{i}+(1-\alpha )\omega_{ij},0\leq \alpha \leq 1.$$ (14)

Where,

Wi = the weight mix;

*ω*_*i*_ = the subjective weight;

*ω*_*ij*_ = the objective weight;

α = 0.65.

### 4.3 Designing the comprehensive early warning evaluation system based on the cloud model

The model of the comprehensive early warning evaluation system of the college simulation enterprise management cockpit is designed based on the principle of cloud modeling. It aims at diagnosing qualitative and quantitative uncertainty transformation of the talent cultivation capacity system in large-scale interdisciplinary simulation platforms.

#### 4.3.1 Constructing the authoritative evaluation set

As common practice shows, three evaluation levels are confirmed according to conventional rating standards, and marked in different colors based on the GE matrix theory. The three levels are: the warning level marked in green, the alert level in yellow, and the danger level in red. A dataset of companies’ three-level indicators is obtained. These companies feature good performance over the years in selected industries.

#### 4.3.2 Transforming the indicator sample dataset into the score one

Based on practical experience, knowledge, and individual opinions about enterprise management of experts on and off campus, statistical evaluation is applied to each third-level indicator of the selected company. By analyzing the aggregation of scatterplot sample group data, the corresponding warning range [*x*_*jmin*_, *x*_*jmax*_] for the sample group is determined, which serves as the warning zone on the cockpit dashboard. Then, considering the positive or negative correlation of the indicators, a score pre-warning interval of [60, 70] is set for positively correlated indicators, and one of [70, 60] set for negatively ones. Moreover, based on two known endpoints (for example, when *x*_*j*_ is a positively correlated indicator, the coordinates of the two endpoints determining the pre-warning zone for the indicator score are [*x*_*jmin*_, 60] and [x_jmax_, 70]), the scoring function for each *x*_*j*_ indicator (corresponding to the third-level dimensional indicator *U*_*mnt*_) is calculated as:

$$y_{j}=ax_{j}+b$$ (15)

Where, a represents the slope of the line. a>0 when the correlation of the indicator is positive and a<0 when the correlation is negative. b is a constant. The value of *y*_*j*_ is then adjusted using an if function: if *y*_*j*_<0, it is set to 0, and if *y*_*j*_>100, it is set to 100.

Next, the corresponding sample group data for each *x*_*j*_ indicator is inputted into the *y*_*j*_ function, and the score dataset is obtained. This process is repeated for the entire sample dataset to get the corresponding scores.

#### 4.3.3 Calculating the standard evaluation cloud

The effective domain H of the evaluation values for each indicator in the score dataset is divided into Z sub-intervals. The *Z*_*j*_-th sub-interval, denoted as (min, max], corresponds to the digital characteristic value of the standard cloud (Ex, En, He). The calculation expressions are as follows:

$$E_{x}=(Zmax+Zmin)/2$$ (16)

$$E_{n}=(Zmax-Zmin)/6$$ (17)

$$H_{e}=K$$ (18)

Where, *Z*_max_ and *Z*_min_ represent the maximum and minimum value obtained from expert ratings.

#### 4.3.4 Calculating the evaluation cloud for the j-th indicator in the sample score dataset

The evaluation cloud for the j-th indicator in the sample score dataset, denoted as *Z*_*j*_(*E*_*xj*_, Enj, Hej), where j = 1, 2,…, n, can be calculated as follows:

$$S^{2}=\frac{1}{n-1}\sum_{i=1}^{n}{\left(x_{i}-\bar{X}\right)}^{2}$$ (19)

$$E_{xj}=\frac{1}{n}\sum_{i=1}^{n}x_{i}$$ (20)

$$E_{nj}=\sqrt{\frac{\pi }{2}}\times \frac{1}{n}\sum_{i=1}^{n}\left|x_{i}-\bar{X}\right|$$ (21)

$$H_{ej}=\sqrt{\left|S^{2}-{E_{nj}}^{2}\right|}$$ (22)

Where,

*S*^2^ = the sample variance;

n = the number of samples;

*x*_*i*_ = the individual values in the sample;

$\bar{X}$ = the sample mean.

#### 4.3.5 Calculating the comprehensive evaluation cloud

To calculate the three numerical characteristics of expected value (E*x*), entropy (E*n*) and hyper-entropy (H*e*), the following formulas are used:

$$Ex=\sum_{j=1}^{n}\left(E_{xj}*Wi\right)$$ (23)

$$En=\sqrt{\sum_{j=1}^{n}\left({E_{nj}}^{2}*Wi\right)}$$ (24)

$$He=\sum_{j=1}^{n}\left(H_{ej}*Wi\right)$$ (25)

Once the comprehensive evaluation cloud is calculated, a comparison is made between the first-level evaluation standard cloud diagram obtained by programming in MATLAB and the generated one. By using the same computational principle in the running of the backend model of the management cockpit, the warning method featuring “observing the corresponding cloud droplet values and membership degrees between the observed indicator cloud diagram and the comprehensive evaluation cloud diagram within the most similar warning interval” is simulated on the dashboard. This can directly show the corresponding warning level of operational status where a simulated company is in.

## 5. Results

### 5.1 Calculating the weight of evaluation model indicators

This paper studies the case of an integrated Guangzhou Huashang College Internship Simulation Platform for business operation of companies. In each period, a total of 54 manufacturing enterprises and 36 distributors provide internship opportunities for students. As the business operation is cycled, the competition is fierce, and companies cannot assess its operational status in a timely manner, it is urgent to put in place a visual tool to track the real-time operation process of the companies, which can provide diagnostic warnings against business risks. Key risks include cash budgeting, bankruptcy, capital structure, operational efficiency, product development, customer management, team building, learning ability and growth. To address the issue in a scientific-based approach, this study adopts a weight mix method to score relevant indicators. A total of 12 experts from the college and 3 experts from Kingdee Precision Information Technology Service Co., Ltd. are invited to score the subjective weighting. Aggregation intervals for scatter plot warnings referenced by objective indicator scoring are selected. Furthermore, the calculation results are adjusted to ensure their validity. It is worth noting that the following indicators correspond to column U in Table 1.

### 5.2 Calculating the weight of the evaluation model indicators using AHP and entropy weight method

The first-level indicators are paired up to evaluate their relative importance by experts and the importance of the indicator weight is scored. The second-level and third-level indicators should do the same. Subjective weights for indicators of three levels are calculated according to Eqs (1) to (5). Next, based on the data of 20 manufacturing enterprises that have performed well in the past three years according to the Guangzhou Huashang College Simulation Platform, combined with practical experience, knowledge, and individual opinions about enterprise management of experts on and off campus, each indicator of the 20 companies in Table 1 is assessed. Based on the positive and negative correlation of the indicators and using scatter plots, the evaluation dataset is obtained and then normalized to calculate the entropy weight according to Eqs (6) to (13) as objective weight. Then, the weight mix is calculated according to Eq (14) (the three types of weights are shown in the left half of Table 2). Finally, the indicators in the left half of Table 2, labeled as “indicator weight (unnormalized) before normalization by U-weight”, are normalized according to the weight of the corresponding first-level indicators to obtain the final data, as are shown in the right half of Table 2.

**Table 2:** Weight calculation of three indicator levels.

Indicator weights (unnormalized) before normalization by U-weight								Indicator weights (normalized) after normalization by U-weight							
First-level	First-level weight	Second-level	Second-level weight	Third-level	Third-level subjective weight	Third-level objective weigh	Third-level composite weight	First-level	First-level weight	Second-level	Second-level weight	Third-level	Third-level subjective weight	Third-level objective weight	Third-level composite weight
U1	0.4072	U11	0.2493	U111	0.2796	0.0603	0.2100	U1	0.4072	U11	0.1015	U111	0.0284	0.0061	0.0206
				U112	0.0262	0.0693	0.0500					U112	0.0027	0.0070	0.0042
				U113	0.0513	0.0465	0.0500					U113	0.0052	0.0047	0.0050
				U114	0.0500	0.0507	0.0500					U114	0.0051	0.0051	0.0051
				U115	0.0600	0.0507	0.0500					U115	0.0061	0.0051	0.0058
				U116	0.0974	0.0508	0.0800					U116	0.0099	0.0052	0.0083
				U117	0.0576	0.0892	0.0700					U117	0.0058	0.0091	0.0070
				U118	0.1580	0.1597	0.1500					U118	0.0160	0.0162	0.0161
				U119	0.0573	0.1179	0.0800					U119	0.0058	0.0120	0.0080
				U1110	0.1037	0.2533	0.1500					U1110	0.0105	0.0257	0.0157
				U1111	0.0589	0.0516	0.0600					U1111	0.0060	0.0053	0.0057
		U12	0.1027	U121	0.2378	0.2403	0.2400			U12	0.0419	U121	0.0101	0.0100	0.0101
				U122	0.1823	0.2049	0.1900					U122	0.0076	0.0086	0.0080
				U123	0.2109	0.2402	0.2200					U123	0.0088	0.0100	0.0092
				U124	0.1915	0.1879	0.1900					U124	0.0080	0.0079	0.0079
				U125	0.1775	0.1267	0.1600					U125	0.0074	0.0054	0.0067
		U13	0.2896	U131	0.2093	0.3871	0.2700			U13	0.1179	U131	0.0247	0.0456	0.0320
				U132	0.3426	0.0880	0.2600					U132	0.0404	0.0104	0.0299
				U133	0.1058	0.0905	0.1000					U133	0.0125	0.0107	0.0119
				U134	0.1231	0.1147	0.1200					U134	0.0145	0.0135	0.0142
				U135	0.0824	0.1177	0.0900					U135	0.0097	0.0139	0.0112
				U136	0.0647	0.1146	0.0800					U136	0.0076	0.0135	0.0096
				U137	0.0721	0.0874	0.0800					U137	0.0085	0.0103	0.0091
		U14	0.1488	U141	0.2217	0.2414	0.2300			U14	0.0606	U141	0.0134	0.0146	0.0138
				U142	0.1823	0.1248	0.1700					U142	0.0110	0.0076	0.0098
				U143	0.1635	0.1161	0.1500					U143	0.0099	0.0070	0.0089
				U144	0.1736	0.1667	0.1700					U144	0.0105	0.0101	0.0104
				U145	0.1727	0.1510	0.1600					U145	0.0105	0.0091	0.0100
				U146	0.0862	0.2000	0.1200					U146	0.0053	0.0122	0.0077
		U15	0.2096	U151	0.2986	0.1889	0.2700			U15	0.0853	U151	0.0255	0.0161	0.0222
				U152	0.2009	0.1952	0.2000					U152	0.0171	0.0167	0.0170
				U153	0.1412	0.1873	0.1600					U153	0.0121	0.0160	0.0135
				U154	0.2115	0.1785	0.1900					U154	0.0181	0.0152	0.0171
				U155	0.1478	0.2501	0.1800					U155	0.0125	0.0213	0.0155
U2	0.0957	U21	0.2000	U211	1.0000	1.0000	1.0000	U2	0.0957	U21	0.0191	U211	0.0191	0.0191	0.0191
		U22	0.4000	U221	1.0000	1.0000	1.0000			U22	0.0383	U221	0.0383	0.0383	0.0383
		U23	0.4000	U231	1.0000	1.0000	1.0000			U23	0.0383	U231	0.0383	0.0383	0.0383
U3	0.2476	U31	0.1972	U311	0.1097	0.1382	0.1200	U3	0.2476	U31	0.0488	U311	0.0054	0.0065	0.0058
				U312	0.1213	0.0872	0.1200					U312	0.0059	0.0043	0.0053
				U313	0.1198	0.0709	0.1200					U313	0.0058	0.0035	0.0050
				U314	0.0867	0.1208	0.0900					U314	0.0042	0.0059	0.0048
				U315	0.1026	0.0606	0.0900					U315	0.0050	0.0030	0.0043
				U316	0.0731	0.1348	0.0900					U316	0.0036	0.0066	0.0047
				U317	0.0799	0.0259	0.0600					U317	0.0039	0.0013	0.0030
				U318	0.1054	0.1143	0.1000					U318	0.0051	0.0056	0.0053
				U319	0.0941	0.1623	0.1100					U319	0.0046	0.0079	0.0057
				U3110	0.1074	0.0850	0.1000					U3110	0.0053	0.0042	0.0049
		U32	0.3134	U321	0.1041	0.0372	0.0900			U32	0.0775	U321	0.0081	0.0029	0.0063
				U322	0.0862	0.2077	0.1300					U322	0.0067	0.0161	0.0100
				U323	0.0602	0.0514	0.0600					U323	0.0047	0.0040	0.0045
				U324	0.0294	0.0466	0.0400					U324	0.0023	0.0036	0.0028
				U325	0.0313	0.0629	0.0400					U325	0.0024	0.0049	0.0033
				U326	0.0726	0.0683	0.0700					U326	0.0056	0.0053	0.0055
				U327	0.0619	0.0587	0.0600					U327	0.0048	0.0046	0.0047
				U328	0.0753	0.1027	0.0900					U328	0.0058	0.0080	0.0066
				U329	0.0390	0.0269	0.0400					U329	0.0030	0.0021	0.0027
				U3210	0.0683	0.0502	0.0600					U3210	0.0053	0.0039	0.0048
				U3211	0.0497	0.0341	0.0400					U3211	0.0039	0.0026	0.0034
				U3212	0.0728	0.0558	0.0700					U3212	0.0056	0.0043	0.0051
				U3213	0.0351	0.0408	0.0300					U3213	0.0027	0.0032	0.0029
				U3214	0.0474	0.0475	0.0400					U3214	0.0037	0.0037	0.0037
				U3215	0.1396	0.0365	0.1100					U3215	0.0108	0.0028	0.0080
				U3216	0.0271	0.0727	0.0300					U3216	0.0021	0.0055	0.0032
		U33	0.2607	U331	0.1462	0.1046	0.1400			U33	0.0646	U331	0.0096	0.0067	0.0086
				U332	0.1234	0.1568	0.1300					U332	0.0080	0.0101	0.0087
				U333	0.0497	0.0442	0.0500					U333	0.0032	0.0029	0.0031
				U334	0.0827	0.0429	0.0700					U334	0.0053	0.0028	0.0044
				U335	0.1058	0.0846	0.0900					U335	0.0068	0.0055	0.0063
				U336	0.0672	0.0704	0.0700					U336	0.0043	0.0045	0.0044
				U337	0.0482	0.0464	0.0800					U337	0.0031	0.0030	0.0031
				U338	0.0819	0.2824	0.1500					U338	0.0053	0.0182	0.0098
				U339	0.0498	0.0429	0.0400					U339	0.0032	0.0028	0.0031
				U3310	0.0617	0.0397	0.0500					U3310	0.0040	0.0026	0.0035
				U3311	0.1834	0.0851	0.1300					U3311	0.0118	0.0055	0.0096
		U34	0.1858	U341	0.2437	0.2457	0.2400			U34	0.0461	U341	0.0112	0.0113	0.0112
				U342	0.2518	0.1961	0.2400					U342	0.0116	0.0090	0.0107
				U343	0.2085	0.1507	0.1900					U343	0.0096	0.0069	0.0087
				U344	0.1524	0.2393	0.1900					U344	0.0070	0.0110	0.0084
				U345	0.1436	0.1682	0.1400					U345	0.0067	0.0079	0.0071
		U35	0.0429	U351	0.5000	0.5000	0.5000			U35	0.0106	U351	0.0053	0.0053	0.0053
				U352	0.5000	0.5000	0.5000					U352	0.0053	0.0053	0.0053
U4	0.1523	U41	0.7000	U411	0.2433	0.1246	0.2000	U4	0.1523	U41	0.1066	U411	0.0259	0.0133	0.0215
				U412	0.2496	0.1038	0.2000					U412	0.0266	0.0111	0.0212
				U413	0.1035	0.2260	0.1500					U413	0.0110	0.0241	0.0156
				U414	0.1197	0.1169	0.1200					U414	0.0128	0.0125	0.0127
				U415	0.1342	0.1298	0.1300					U415	0.0143	0.0137	0.0141
				U416	0.0746	0.1349	0.1000					U416	0.0080	0.0144	0.0102
				U417	0.0751	0.1640	0.1000					U417	0.0080	0.0175	0.0113
		U42	0.3000	U421	0.7000	0.7100	0.7000			U42	0.0457	U421	0.0320	0.0324	0.0321
				U422	0.3000	0.2900	0.3000					U422	0.0137	0.0133	0.0136
U5	0.0972	U51	0.1973	U511	1.0000	1.0000	1.0000	U5	0.0972	U51	0.0192	U511	0.0192	0.0192	0.0192
		U52	0.5113	U521	0.6429	0.6302	0.6400			U52	0.0497	U521	0.0319	0.0313	0.0317
				U522	0.1342	0.1189	0.1200					U522	0.0067	0.0059	0.0064
				U523	0.0498	0.0394	0.0500					U523	0.0025	0.0020	0.0023
				U524	0.1731	0.2115	0.1900					U524	0.0086	0.0105	0.0093
		U53	0.2914	U531	0.4156	0.3826	0.4100			U53	0.0283	U531	0.0118	0.0108	0.0115
				U532	0.3579	0.3571	0.3600					U532	0.0101	0.0101	0.0101
				U533	0.2265	0.2603	0.2300					U533	0.0064	0.0074	0.0067
total	1		5		18	18	18		1		1		1	1	1

Regarding specific calculation, this study adopts the subjective weight solving process of 11 three-level indicators of financial early warning U11 as an example to illustrate the consistency test of AHP, as is shown in Table 3.

**Table 3:** Calculation of the subjective weights of 11 three-level indicators of financial early warning U11.

Objective	U111	U112	U113	U114	U115	U116	U117	U118	U119	U1110	U1111	Subjective weight	Normalized weight
U111	1	5	6	6	6	3	5	2	4	3	6	0.2796	0.0284
U112	1/5	1	1/4	1/3	1/5	1/2	1/4	1/3	1/2	1/2	1/3	0.0262	0.0027
U113	1/6	4	1	2	1/3	1/2	1	1/3	1	1/2	1/2	0.0513	0.0052
U114	1/6	3	1/2	1	2	1/2	1	1/3	1/3	1/2	1	0.0500	0.0051
U115	1/6	5	3	1/2	1	1/2	1	1/3	1	1/2	1	0.0600	0.0061
U116	1/3	2	2	2	2	1	2	1/2	2	1	2	0.0974	0.0099
U117	1/5	4	1	1	1	1/2	1	1/3	1	1/2	1	0.0576	0.0058
U118	1/2	3	3	3	3	2	3	1	3	2	3	0.1580	0.0160
U119	1/4	2	1	3	1	1/2	1	1/3	1	1/4	1	0.0573	0.0058
U1110	1/3	2	2	2	2	1	2	1/2	4	1	2	0.1037	0.0105
U1111	1/6	3	2	1	1	1/2	1	1/3	1	1/2	1	0.0589	0.0060

According to important of the paired indicators, the 11 third-level indicators of financial warning U11 in Table 3 are scored, and subjective weights are calculated. According to Table 4, it is found that the RI value is 1.5135. Then, using Eq (5), CR is calculated as 0.0522, which is less than 0.1, indicating that the dataset has acceptable consistency. This example demonstrates that the overall subjective weighting of experts complies with the consistency test. Based on the left half of Table 2, the subjective weights of the indicators are multiplied by the first-level indicator weight of U1 (0.4072) and then by the second-level indicator weight of U11 (0.2493). By doing so, the normalized weights for this group are generated, aligning with the weights of the 11 indicators that belong to U11 in the right half of Table 2. This accounts for how sample data are calculated and normalized.

**Table 4:** Average random consistency index RI.

**M**	1	2	3	4	5	6	7	8	9
**RI**	0	0	0.5275	0.8824	1.1075	1.2468	1.3394	1.4039	1.4511
**M**	10	11	12	13	14	15	16	17	18
**RI**	1.4863	1.5135	1.5363	1.5541	1.5712	1.5838	1.5962	1.6051	1.6135

### 5.3 The evaluation of the comprehensive cloud model warning

#### 5.3.1 Determining the criteria for the evaluation of the cloud model comprehensive early warning for enterprise management cockpit

**Fig 4:**
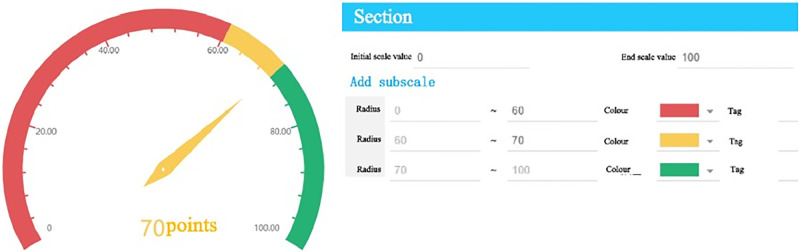
An example of the warning area division on the management dashboard of internship simulation platform for enterprise operation at Guangzhou Huashang College.

There are three enterprise warning levels for the college simulation platform, which are categorized using a digital grading system, where higher values indicate greater safety. Based on the actual operation of enterprises on the simulation platform, each warning level ranges from (0–60], (60–70], and (70–100] respectively. In this case, the value of K isset as 0.1, and a dial is set in the Kingdee Light Analysis Software, as shown in Fig 4. In this study, He is determined as 0.1.

Based on the characteristics of the warning intervals in Fig 4, and in accordance with Eqs (16) to (18), the cloud model for the warning levels are calculated, as shown in Table 5.

**Table 5:** Cloud model for enterprise warning levels on the college simulation platform.

Warning level	Score interval	Standard cloud
Dangerous	(0–60]	(30, 10, 0.1)
General	(60–70]	(65, 1.67, 0.1)
Good	(70–100]	(85, 5, 0.1)

**Fig 5:**
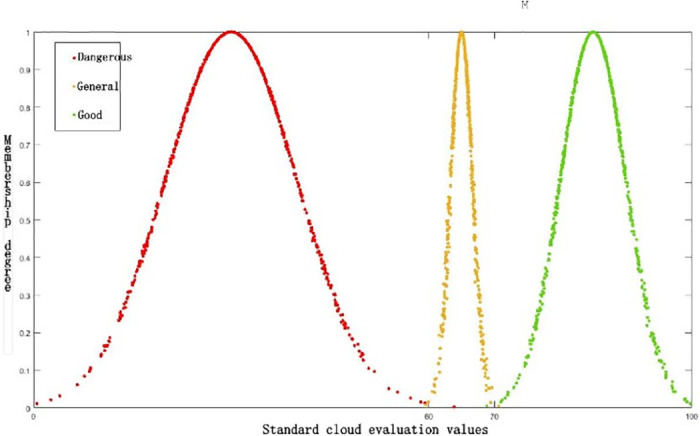
Standard cloud image of enterprise management cockpit.

Then, the standard cloud of early warning level is programmed by MATLAB to obtain the standard cloud map, as shown in Fig 5.

#### 5.3.2 Computing the evaluation cloud set for all sample indicators

According to Eqs (15), (19) to (22), the evaluation cloud set (Exj, Enj, Hej) of scores for each level indicators of the sample enterprises are calculated, as presented in Table 6.

**Table 6:** Evaluation cloud for indicators at each level.

Third-level indicators	Exj	Enj	Hej
U111	68.7	4.32	0.07
U112	68.7	4.11	0.88
U113	67.8	5.05	0.34
U114	69.9	1.83	0.48
U115	74.6	5.47	1.47
U116	80.4	14.26	3.43
U117	76.5	10.34	1.74
U118	70.8	11.45	3.64
U119	69.1	9.17	1.98
U1110	73.8	12.97	2.22
U1111	68.8	6.37	1.4
U121	73.3	8.24	1.79
U122	79	10.98	3.55
U123	73.3	8.24	1.79
U124	74.3	12.63	5.27
U125	74	13.29	5.01
U131	72.6	13.76	2.78
U132	72.6	13.76	2.78
U133	84.7	17.91	1.77
U134	84.5	14.18	2.73
U135	83.4	12.21	3.08
U136	67.2	3.57	0.86
U137	62	5.25	0.9
U141	64.9	5.44	2.11
U142	79.8	10.42	2.44
U143	67.7	10.5	3.12
U144	83.4	12.21	3.08
U145	82.9	19.43	7.9
U146	70.7	5.48	0.76
U151	66.8	5.78	1.39
U152	68.3	8.91	2.38
U153	66.8	5.94	1.62
U154	67.4	6.05	1.79
U155	70.3	6.67	2.02
U211	76.6	5.16	1.55
U221	76.6	5.64	1.25
U231	75.8	4.14	0.84
U311	66.5	13.16	4.2
U312	65.5	8.12	1.38
U313	73.4	11.89	2.91
U314	66.7	2.91	1.04
U315	70	6.27	1.23
U316	69	7.02	1.72
U317	67.3	6.71	5.19
U318	68.1	5.8	1.66
U319	68.8	7.83	3.01
U3110	68.7	6.89	2.48
U321	73	14.16	0.52
U322	79.7	14.64	2.5
U323	71.4	10.83	2.67
U324	70.7	6.7	1.22
U325	70.7	6.7	1.22
U326	67.9	10.57	3.63
U327	63.9	14.95	1.92
U328	67.5	6.27	2.52
U329	64.2	6.35	3.42
U3210	77.2	12.45	1.68
U3211	74.9	5.77	0.24
U3212	69.9	5.45	1.67
U3213	69.9	8.69	2.71
U3214	71.8	9.71	3.07
U3215	67.3	4.78	1.18
U3216	78.5	12.28	0.77
U331	68.6	5.69	1.92
U332	69.3	7.15	1.86
U333	82.9	18.59	13.72
U334	83.3	19.84	13.16
U335	94.2	5.01	0.34
U336	89.5	10.83	1.72
U337	73.3	4.24	3.23
U338	70.8	12.36	3.77
U339	66.1	13.37	14.12
U3310	68.3	13.86	14.15
U3311	75.3	20.66	6.39
U341	66.2	7	2.89
U342	67.6	8.51	3.48
U343	67.5	5.28	0.84
U344	67.3	12.47	4.14
U345	78.8	2.54	0.67
U351	77.6	6.64	1.07
U352	78.9	6.56	2.22
U411	67.7	6.84	2.07
U412	67	5.59	1.25
U413	73	8.77	3.57
U414	71.6	7.82	1.24
U415	79.1	4.04	0.44
U416	63.3	7.38	3.47
U417	76.2	6.42	1.7
U421	78.3	5.23	0.99
U422	72.9	6.4	1.2
U511	79.6	2.89	0.66
U521	78.1	5.04	1.55
U522	75.2	4.47	0.94
U523	76.8	6.74	0.49
U524	75.2	6.14	1.33
U531	76.2	4.74	0.65
U532	75.6	5.76	0.41
U533	72.4	4.07	1.14

#### 5.3.3 Determining the evaluation cloud for enterprises on the college simulation platform

Referring to Table 6, the total evaluation cloud Z (73.2, 9.12, 2.18) for the three-level indicators of enterprises on the Guangzhou Huashang College Simulation Platform was obtained by employing Eqs (23) to (25).

#### 5.3.4 Determination of the warning level for enterprises on the simulation platform

**Fig 6:**
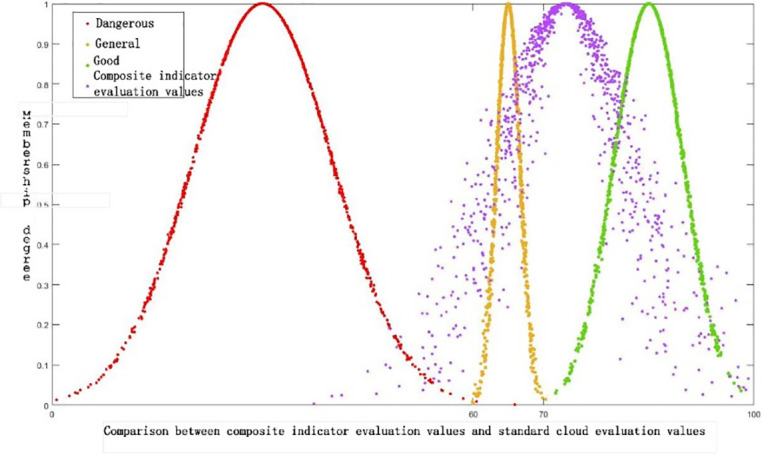
Comparison between composite indicator evaluation values and standard cloud evaluation values.

The evaluation cloud map Z (73.2, 9.12, 2.18) for the early warning levels are drawn based on the comprehensive evaluation cloud of enterprises on the Guangzhou Huashang College Simulation Platform. It is then compared with the standard cloud map. By programming the standard cloud map using MATLAB, the results are shown in Fig 6. For the warning level of “General” and “Good”, the comprehensive cloud map is further analyzed to study its numerical features. It is determined that the cloud map is most similar to the “Good” standard one. Therefore, the safety evaluation level for enterprises on the Guangzhou Huashang College Simulation Platform is determined to be “Good”. This indicates that these enterprises can operate in a good state; however, continuous improvement is needed in key areas such as the main business profit margin U145, operating leverage ratio U317, current ratio U333, quick ratio U334, cash ratio U339, and cash flow ratio U3310, in case of a shift to the dangerous level.

## 6. Main conclusions and recommendations

### 6.1 Main conclusions

Firstly, the enterprise management cockpit is a tool that utilizes data visualization and information technology to present key performance indicators of an organization. It allows the management personnel to access key data on an integrated platform, so as to better understand the condition and development of the enterprise. Researches have shown that this management approach is of great significance in improving the overall operational management level of enterprises on the college simulation platform. It helps the management personnel to gain a better understanding of the business through macro system and micro indicators and find risks in advance, and supports their decision-making.

Secondly, an evaluation indicator system for the operational safety of enterprises is established based on five management dimensions: integration of business and finance, customers, internal operations, products, and teams. This system reflects various factors necessary to assess the safety of enterprises on the simulation platform. By using the subjective weights derived from the AHP and the objective weights calculated through the entropy weight method, and the weight mix of each indicator is obtained in proportion. This approach reflects the importance of each indicator in an objective way and provides reliable data for the comprehensive warning evaluation of the college simulation enterprise management cockpit cloud model. It bridges the gap in the digitalization of the platform throughout the warning diagnosis process.

Thirdly, the application of cloud models in establishing a safety evaluation model for simulation platform enterprises enables a comprehensive understanding of the overall operational safety. It allows real-time feedback on the warning levels under different operational conditions of enterprises.

### 6.2 Recommendations

The enterprise management cockpit of the university management simulation platform can provide comprehensive and real-time operation monitoring and decision support data query, analysis, and early warning assistance. Its limitations lie in the complexity of data integration, limitations on model accuracy, and system scalability. In response to these limitations, further research areas can focus on empirical verification of the multi indicator scientific comprehensive system of combination and cloud models, and compare the latest AI functions and automation technologies to improve data integration efficiency, optimize model accuracy, and explore more flexible and scalable system architectures to break through bottlenecks in this field and promote further development of research.
